# A microRNA signature profile in EBV^+^ diffuse large B-cell lymphoma of the elderly

**DOI:** 10.18632/oncotarget.2952

**Published:** 2014-12-10

**Authors:** Tathiana Azevedo de Andrade, Adriane Feijo Evangelista, Antonio Hugo Froes Campos, Wagner Augusto Poles, Natalia Morais Borges, Claudia Malheiros Coutinho Camillo, Fernando Augusto Soares, Jose Vassallo, Roberto Pinto Paes, Maria Claudia Zerbini, Cristovam Scapulatempo, Antonio Correa Alves, Ken H. Young, Gisele Wally Braga Colleoni

**Affiliations:** ^1^ Universidade Federal de Sao Paulo, UNIFESP, Sao Paulo, Brazil; ^2^ Hospital de Cancer de Barretos, Barretos, Brazil; ^3^ A.C. Camargo Cancer Center, Sao Paulo, Brazil; ^4^ Universidade de Campinas, Campinas, Brazil; ^5^ Santa Casa de Misericordia de Sao Paulo, Sao Paulo, Brazil; ^6^ Faculdade de Medicina da Universidade de Sao Paulo, Sao Paulo, Brazil; ^7^ Department of Hematopathology, MD Anderson Cancer Center, Houston, Texas, USA

**Keywords:** DLBCL, EBV, elderly, microRNA

## Abstract

Currently, there is no characteristic microRNA (miRNA) expression pattern in Epstein-Barr virus^+^ diffuse large B-cell lymphoma of the elderly (EBV^+^DLBCLe). This study aims to characterize a signature profile and identify miRNAs that can be used as biomarkers and alternative therapeutic targets for EBV^+^DLBCLe. Seventy-one DLBCL patients aged 50 years and older were included and four EBV^+^ and four EBV– samples were analyzed in two miRNA array platforms (pilot study). A larger multicenter cohort (29 EBV^+^DLBCLe and 65 EBV–DLBCL patients) was used to validate the results by real-time polymerase chain reaction. In the pilot study, 9% of DLBCL were EBV^+^DLBCLe by *in situ* hybridization. In multicenter study, EBV^+^DLBCLe group showed a predominance of non-germinal center B-cell origin. Overall survival duration of EBV^+^DLBCLe was significantly inferior to that of EBV–DLBCL patients. We found 10 deregulated miRNAs in the two groups, but only seven were statistically different. We confirmed overexpression of hsa-miR-126, hsa-miR-146a, hsa-miR-146b, hsa-miR-150, and hsa-miR-222 and underexpression of hsa-miR-151 in EBV^+^DLBCLe cases compared to EBV–DLBCL cases. Hsa-miR-146b and hsa-miR-222 showed high specificity for identifying EBV^+^DLBCLe. The present study proposed a miRNA signature for EBV^+^DLBCLe and our findings suggest that hsa-miR-146b and hsa-miR-222 could be biomarkers and therapeutic targets.

## INTRODUCTION

Epstein-Barr virus^+^ diffuse large B-cell lymphoma of the elderly (EBV^+^DLBCLe) is considered a provisional entity in the latest World Health Organization classification system[1]. It affects individuals older than 50 years with no prior documented immunodeficiency. This disorder has an unfavorable clinical course, even after anthracycline-based chemotherapy [2]. It is linked to EBV infection, and its physiopathological characteristics are related to the presence of the virus itself, senescence, and immunological deterioration [3]. It can arise as the result of EBV latency protein expression, interaction with host factors, and epigenetic mechanisms of gene regulation, including microRNA (miRNA) expression [4].

MiRNAs are small RNAs with 18 to 25 nucleotides that result from cleavage of a longer non-coding RNA; they can interfere with gene regulation by binding to the complex of RNA-induced silencing, causing repression of translation and influencing differentiation, proliferation, cell survival, and apoptosis [5]. On the basis of current findings reported in the literature, viral miRNAs appear to have a small role in EBV+DLBCLe [6]. However, many of them are homologous with human miRNAs, which may explain the highly aggressive behavior of this disease. Supporting this hypothesis, the results of recent studies indicate that miRNAs from EBV are directly related to oncogenesis in several biological processes such as B-cell activation, oxidative stress response, cytokine-mediated inflammation, transcription pathway activation, and apoptosis inhibition [6,7].

Although miRNA expression profiling was recently described for EBV^+^DLBCL in general [8] or using different methodologies (PAR-CLIP) in lymphoblastoid cell lines [9], to our knowledge, no characteristic pattern of miRNA expression has been identified for elderly patients, i. e., EBV^+^DLBCLe. Therefore, in this retrospective study, we characterized a signature profile for this entity and identified unique miRNAs that can be used as biomarkers and alternative therapeutic targets for EBV^+^DLBCLe.

## RESULTS

ISH for EBV revealed that 9% of DLBCL cases (6 patients) in the pilot study were EBV^+^DLBCLe. This was subsequently expanded with 23 new cases (multicenter study). The clinical features of all patients included in the study (pilot and multicenter) are shown in Table 1.

**Table 1:** Clinical features and results of immunohistochemical classification, according to the Hans (2004) and Salles (2011) algorithms, in 94 DLBCL patients (pilot and multicenter) aged 50 years or older, evaluated according to positivity for EBV by ISH

Characteristic	EBV+DLBCLe (n=29)	EBV−DLBCL (n=65)	P value*
Median age, years (range)	67 (51-88)	63 (50-85)	
Sex, n (%)			
Male	12 (41)	29 (45)	0.7701
Female	17 (59)	36 (55)	
Ann Arbor, n (%)			
I-II	10 (37)	25 (39)	0.898
III-IV	17 (63)	40 (62)	
N/A	2	0	
B symptoms, n (%)			
𠀲No	16 (59)	20 (31)	**0.0108**
𠀲Yes	11 (41)	45 (69)	
𠀲N/A	2	0	
Extranodal sites, n (%)			
No	23 (79)	39 (62)	0.098
Yes	6 (21)	24 (38)	
N/A	0	2	
IPI, n(%)			
≤	12 (55)	28 (45)	0.449
>2	10 (46)	34 (55)	
N/A	7	3	
Hans algorithm (2004), n (%)			
GCB	7 (28)	28 (52)	**0.0472**
Non-GCB	18 (72)	26 (48)	
NC	4	11	
Salles algorithm group (2011), n (%)			
1	5 (24)	5 (9)	
2	3 (14)	11 (19)	
3	10 (48)	28 (48)	
4	3 (14)	15 (25)	
NC	8	6	
Salles classification group (2011), n (%)			
1 + 2	8 (38)	16 (27)	0.3459
3 + 4	13 (62)	43 (73)	
NC	8	6	

N/A = not available

NC = not classifiable

In the EBV^+^DLBCLe group (n=29), patients were aged 51 to 88 years (median, 67 years). There were 12 male and 17 female patients; 63% had advanced Ann Arbor stage disease, 55% had an IPI score ≤2, 41% had B symptoms, and 21% presented with extranodal involvement (gastrointestinal tract, skin, and breast).

In the EBV–DLBCL group (n=63), the median age was 63 years (range, 50 to 85 years). There were 29 males and 36 females; 62% had advanced Ann Arbor stage disease, 69% had B symptoms, and 38% had extranodal involvement (gastrointestinal tract, skin, breast, lung, kidney, testis, and thyroid). The IPI was ≤2 in 45% of cases. The comparison of the two groups revealed no statistical difference in relation to the clinical characteristics described above, except for the presence of B symptoms, which was more prevalent in EBV–DLBCL patients (69.3% versus 40.7%, p=0.0108, chi-square test).

All 94 DLBCL patients in the multicenter group were evaluated using the Hans algorithm (Table 1). In the EBV^+^DLBCLe group, 28% of cases had germinal B-cell (GCB) and 72% non-GCB; in the EBV–DLBCL group, 52% of cases had GCB and 48% non-GCB (p=0.0472, chi-square test). CD30 were positive in 7% of cases.

In the EBV^+^DLBCLe group, Bcl-2 was positive in 54% and Ki-67 in 67% of cases. Combining the IPI with these two immunohistochemical markers, we classified the cases according to the Salles algorithm into groups 1 (24%), 2 (14%), 3 (48%), and 4 (14%). In the EBV–DLBCL group, Bcl-2 was positive in 57% and Ki-67 in 90% of cases. These cases were classified into groups 1 (9%), 2 (19%), 3 (48%), and 4 (25%). In both the EBV^+^DLBCLe and EBV–DLBCL groups, more cases were in the poor prognosis subgroups. We found no statistical difference between groups when we combined the Salles algorithm groups (1+2 and 3+4) (p=0.3459, chi-square test).

34% of EBV^+^DLBCLe cases (multicenter study) were classified as monomorphic and 66% of cases as polymorphic. The polymorphic cases were also classified as canonical large B-cell-type (31%), polymorphic lymphoproliferative disorder-like variant (27%) and Hodgkin lymphoma-like variant (8% of total EBV^+^ cases) according to Montes-Moreno's subclassification scheme [20]. We found 62.5% of positivity for LMP1.

**Figure 1:**
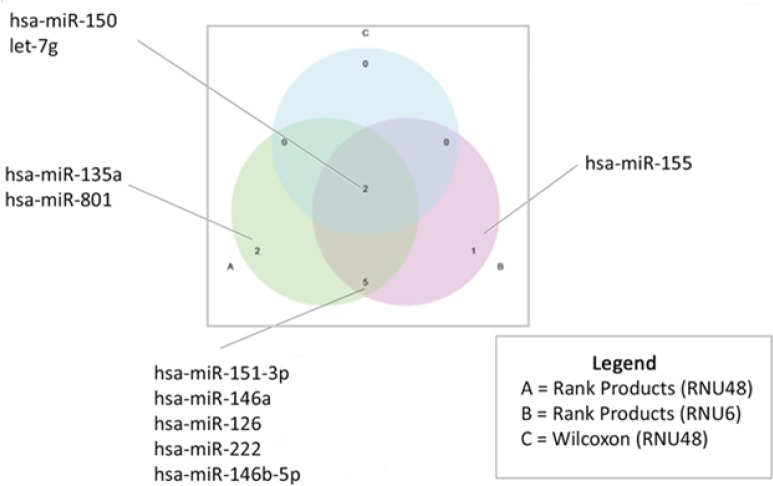
Schematic representation of miRNAs that were differentially expressed between the four cases of EBV^+^DLBCLe and four cases of EBV^−^DLBCL, considering two normalizing miRNAs (RNU48 and U6) and two different statistical methods (Wilcoxon rank and products) (A) miRNAs that were identified as differentially expressed by the rank products method, using the normalizer miRNA RNU48. (B) miRNAs that were identified as differentially expressed by the rank products method using the normalizer miRNAU6. (C) miRNAs were assessed by the Wilcoxon statistical method using the normalizer miRNA RNU48. The areas of intersection display the number of miRNAs that were identified as differentially expressed, assessed simultaneously using more than one method or more than one normalizer. These seven miRNAs were evaluated in the next steps.

In the pilot study, we selected four EBV^+^DLBCLe and four EBV–DLBCL samples matched by age, sex, stage, and IPI and analyzed them in miRNA array platforms. The decision of the number of cases to be used in this phase of the study took in consideration costs and quality of the RNA samples. The analysis revealed that some miRNAs were differentially expressed between the groups (Figure 1). The areas of intersection show the number of miRNA candidates for validation in the next step, which were simultaneously evaluated using two statistical methods (rank products and Wilcoxon) or more than one normalizer (RNU48 and U6).

We found 10 deregulated miRNAs among the two groups. However, only seven miRNAs were statistically significantly different and were included in the miRNA signature profile proposal that was validated in the multicenter cohort. Among them, hsa-let-7g, hsa-miR-126, hsa-miR-146a, hsa-miR-146b, hsa-miR-150 and hsa-miR-222 were overexpressed in EBV^+^DLBCLe compared to in EBV–DLBCL, whereas miR-151 was underexpressed.

The results, after validation, demonstrated that hsa-miR-126 was overexpressed in 76% (median, 2.14 *versus* 0.14, p<0.0001), hsa-miR-146a in 62% (median, 1.92 *versus* 0.29, p<0.0001), hsa-miR-146b in 52% (median, 1.51 *versus* 0.11, p<0.0001), hsa-miR-150 in 97% (median, 20.54 *versus* 2.56, p<0.0001), and hsa-miR-222 in 31% (median, 0.68 *versus* 0.08, p<0.0001, Mann-Whitney test) of EBV^+^DLBCLe cases compared with EBV–DLBCL cases. Hsa-miR-151 was underexpressed in 86% (median, 0.30 *versus* 0.09, p<0.0015, Mann-Whitney test) and hsa-let-7g in 72.4% of EBV^+^DLBCLe cases. In the validation, has-let-7g revealed opposite result from pre-validation and showed no difference in behavior between the two groups (median, 0.41 *versus* 0.39, p=0.9053, Mann-Whitney test) (Table 2).

**Table 2:** Validation of human miRNAs that were differentially expressed in 29 EBV+DLBCLe cases and 65 cases EBV–DLBCL cases by quantitative real-time PCR and summary of the main targets of hsa-miR-146b (through its counterpart EBV-miR-BART3) and hsa-miR-222, as identified by GeneCards (http://www.genecards.org) Gene Reference Into Function (GeneRIF) (http://www.ncbi.nlm.nih.gov/gene)

MiRNA	miRNA overexpression (cut-off >1.5) in EBV+DLBCLe, n(%)	Median relative miRNA (EBV+DLBCLe *vs.* EBV−DLBCL)	P value*
hsa-let-7g	4 (14)	0.39 *vs.* 0.41	0.9053
hsa-miR-126	22 (76)	2.14 *vs.* 0.14	**<0.0001**
hsa-miR-146a	18 (62)	1.92 *vs.* 0.29	**<0.0001**
hsa-miR-146b	15 (52)	1.51 *vs.* 0.11	**<0.0001**
hsa-miR-150	28 (97)	20.54 *vs.* 2.56	**<0.0001**
hsa-miR-151	01 (3)	0.30 *vs.* 0.09	**0.0015**
hsa-miR-222	09 (31)	0.68 *vs.* 0.08	**<0.0001**

MiRNA	Target	Function
hsa-miR-146b	*INST6*	Tumor suppression
hsa-miR-222	*PTEN*	Tumor suppression
hsa-miR-222	*CDKNJB (p27), CDKNJC*	Cell cycle regulation
hsa-miR-222	*STAT5A*	Transcription
hsa-miR-222	*FOS, KIT*	Oncogene
hsa-miR-222	*ICAMJ*	Adhesion molecule
hsa-miR-222	*SOD2*	Oxidative stress

**Figure 2:**
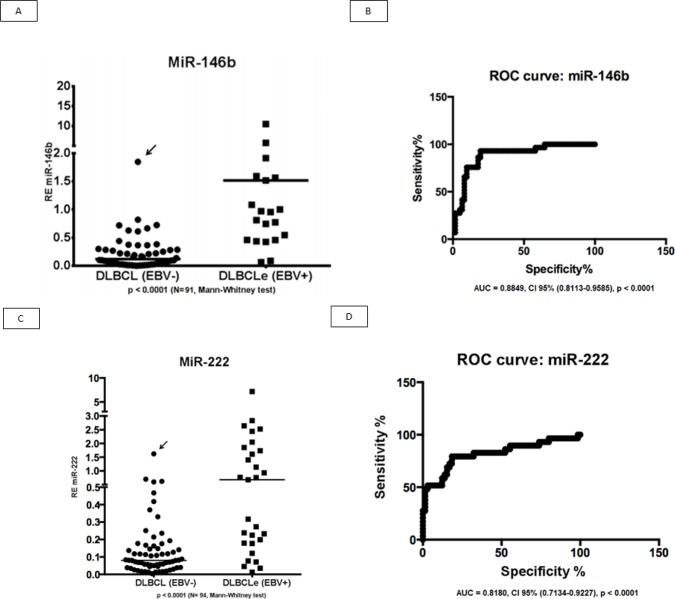
(A) Relative expression (RE) of hsa-miR-146b in EBV+DLBCLe and EBV–DLBCL cases by qPCR. hsa-miR-146b was overexpressed in EBV^+^DLBCLe compared to in EBV^−^DLBCL (median, 1.51 vs. 0.11, p<0.0001, Mann-Whitney test). Note that only one EBV^−^DLBCL case (arrow) had a fold change value higher than 1.5. (B) An ROC curve of hsa-miR-146b showed a sensitivity of 65.2%, specificity of 91.4%, positive predictive value of 75%, and negative predictive value of 86.9%in EBV^+^DLBCLe cases compared to in EBV^−^DLBCL cases. (C) The relative expression (RE) of hsa-miR-222 was determined in EBV^+^DLBCLe and EBV^−^DLBCL cases. hsa-miR-222 was overexpressed in EBV^+^DLBCL compared to in EBV^−^DLBCL (median, 0.68 vs. 0.08, p<0.0001, Mann-Whitney). Note that there was only one case (arrow) in EBV^−^DLBCL with a fold change value higher than 1.5. (D) An ROC curve of hsa-miR-222 showed a sensitivity of 23%, specificity of 98.5%, positive predictive value of 90%, and negative predictive value of 76.2% in EBV^+^DLBCLe compared to in EBV^−^DLBCL.

We evaluated two miRNAs as potential biomarkers for EBV^+^DLBCLe after we evaluated the graphical representation of relative expression in both groups: hsa-miR-146b and hsa-miR-222. We used the fold change of 1.5 as the cut-off to compare EBV^+^DLBCLe and EBV–DLBCL cases and found that miR-146b had a sensitivity of 65.2%, specificity of 91.4%, positive predictive value of 75%, negative predictive value of 86.9%, and area under the curve of 0.8849 (ROC curve) (Figure 2, *A* and *B*). Despite being overexpressed in less than one-third of EBV^+^DLBCLe cases (Figure 2 *C*) hsa-miR-222 had a sensitivity of 31%, specificity of 98.5%, positive predictive value of 90%, negative predictive value of 76.2%, and area under the curve of 0.8180 (ROC curve) using the same cut-off of 1.5 (Figure 2 *D*). When hsa-miR-222 was analyzed, we noted that the EBV^+^DLBCLe group presented two subpopulations with different behaviors (Figure 2 *C*): among those with increased expression of has-miR-222, 100% of cases were non-GCB and were classified as having the worst prognosis according to Salles algorithm; 86% had Ann Arbor stage III or IV, and 71% had an IPI higher than 2.

The EBV^+^DLBCLe subpopulation with underexpression of hsa-miR-222 was classified as follows: 67% were non-GCB; 55% were group 1 or 2 of the Salles algorithm, 60% had Ann Arbor stage III and IV, and 72% had IPI ≤2. The chi-square test was applied to each of the parameters mentioned above, and the EBV^+^DLBCLe group, with increased expression of hsa-miR-222, was predominantly rated as group 3 or 4 of the Salles algorithm (p=0.0245).

**Figure 3:**
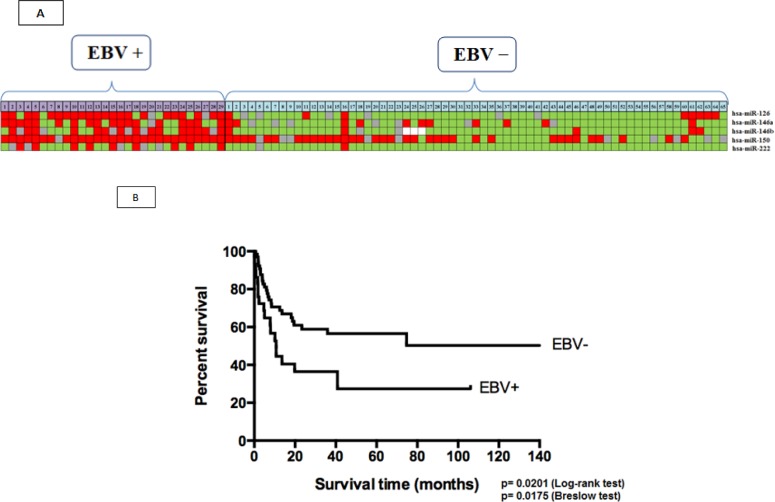
(A) Signature profiling. The expression of miRNAs in patients with EBV^+^DLBCLe and EBV^−^DLBCL was determined in the final multicenter study by qPCR. Red indicates overexpression (>1.5), gray normoexpression (0.66 to 1.5), green underexpression (<0.66), and white information not available. (B) OS curves of patients included in the study according to the ISH result.

Figure 3 *A* summarizes the results of qPCR of the five most relevant miRNAs in the study, excluding hsa-let-7g (due to a lack of statistical significance) and hsa-miR-151 expression (due to decreasing overall expression in both groups). Using the colors red (overexpression >1.5), gray (normoexpression, 0.66 to 1.5), and green (underexpression <0.66), we simulated the signature profile of the miRNAs in EBV^+^DLBCLe.

The median OS duration of the 94 patients in the multicenter study was 40.77 months. We performed a survival analysis to compare the EBV^+^DLBCLe and EBV– groups by IPI score (low, intermediate, and high), cellular origin (GCB *versus* non-GCB), and Salles classification and prognostic subgroups. The OS of EBV^+^DLBCLe patients was lower than that of EBV–DLBCL patients (log-rank test, p=0.0201; Breslow test, p=0.0175) (Figure 3*B*). The difference between the two groups (EBV^+^ versus EBV^−^) OS was kept when only CHOP-treated patients were analyzed, excluding those who received R-CHOP (13 cases) (log-rank test, p = 0.0334). No other differences in survival were found, including the seven studied miRNAs.

**Figure 4:**
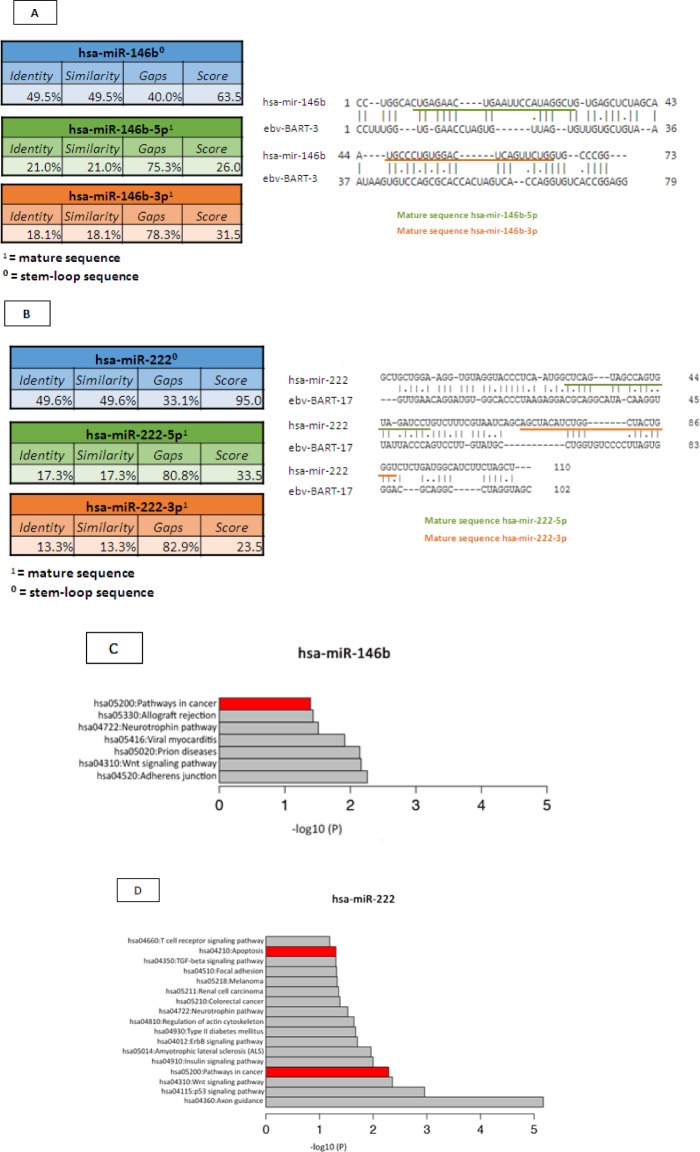
(A) Homologous features between hsa-miR-146b and EBV-miR-BART3, according to the Needleman-Wunsch algorithm. (B) Homologous features between hsa-miR-222 and EBV-miR-BART17, according to the Needleman-Wunsch algorithm. (C) We determined which pathways of hsa-miR-146b were involved in oncogenesis according to DAVID version 6.7 (http://david.abcc.ncifcrf.gov) and the KEGG Pathway Database (http://www.genome.jp/kegg/pathway.html). The targets related to cancer pathways are identified in red: PTGS2, EGLN3, FZD1, SMAD4, RUNX1T1, CDH1, APPL1, CCDC6, NRAS, LAMB3, WNT3, RAC2, RHOA, PDGFRB, RARA, RARB, FAS, TRAF6, and FN1.D) We determined which hsa-miR-222 pathways are involved in oncogenesis and apoptosis, according to DAVID version 6.7 (http://david.abcc.ncifcrf.gov) and the KEGG Pathway Database (http://www.genome.jp/kegg/pathway.html). The oncologic targets were: TRAF2, FGF5, MITF, TFG, KIT, MMP1, ARNT, FOS, BCL2, PAX8, RHOA, RALA, AXIN2, PIK3R1, DVL2, CYCS, IGF1, FZD3, CDK6, MAPK10, RAD51, CTNNA2, MAPK1, CRKL, CDKN1B, ETS1, and MDM2. The apoptosis targets were TRAF2, PRKAR2A, BCL2, IL1RAP, CYCS, PPP3R1, APAF1, ATM, and PIK3R1. Both are represented in red.

Once we had identified the miRNAs that were differentially expressed between the EBV^+^DLBCLe and EBV–DLBCL groups and considering that some EBV miRNAs are homologous with human miRNAs, we performed a homology blast of the seven selected human miRNAs with 44 virus miRNAs, according to the methods of Babu and colleagues [18]. We identified homologous features between two selected human miRNAs and EBV miRNAs: hsa-miR-146b was homologous with EBV-miR-BART3 (score 63.5 and 49.5% identity), and hsa-miR-222 was homologous with EBV-miR-BART17 (score 95 and 49.6% identity). The homology alignments according to the Needleman-Wunsch algorithm and the identities and scores are represented in Figure 4 *A* and B, representing the stem-loop and the mature 5p and 3p sequences of the two relevant miRNAs.

To analyze the pathways and targets of hsa-miR-146b, we used its viral counterpart, EBV-miR-BART3, as a reference. This viral miRNA inhibits the tumor suppressor *INTS6* (NM_001039937) gene, also called *DICE-1* [21] (these data were obtained from the information available at Gene Reference Into Function [GeneRIF] [http://www.ncbi.nlm.nih.gov/gene]). Another target of EBV-miR-BART3 is IPO7, which is known for its involvement in inflammatory processes [21]. Using the GeneCards tool (http://www.genecards.org) to predict targets of hsa-miR-222, we obtained the following results (protein*, identifier search targets by miRTarBase): CORO1A (MIRT005791), ESR1 (MIRT005321), SELE (MIRT005715), PTEN (MIRT005586), MMP1 (MIRT000136), BBC3 (MIRT005369), STAT5A (MIRT000018), SOD2 (MIRT000135), KIT (MIRT001779), CDKN1B (MIRT000131), CDKN1C (MIRT000719), SSSCA1 (MIRT005790), PPP2R2A (MIRT003191), FOS (MIRT004485), TP53 (MIRT005786), TCEAL1 (MIRT005792), FOXO3 (MIRT000433), and ICAM1 (MIRT004595).

According to the information available at GeneRIF, we found eight possible targets for has-miR-222 that were common to both search methods, namely, CDKN1B (p27), CDKN1C (p57), FOS, ICAM1, KIT, PTEN, SOD2, and STAT5A (identified in the GeneCards list above). Hsa-miR-222 interferes with important targets related to oncogenesis, including the tumor suppressor PTEN. We also found targets involved in cell cycle regulation, such as CDKN1B (p27) and CDKN1C; cell transcription, such as STAT5A; oncogenes, such as FOS and KIT; adhesion molecules, such as ICAM1; and oxidative stress, such as SOD2 (http://string-db.org). Its miRNA viral counterpart, EBV-miR-BART17, has as its principal target BCLAF1, which blocks apoptosis.

* BBC3: BCL2 binding component 3; CDKN1B: cyclin-dependent kinase inhibitor 1B; CDKN1B: cyclin-dependent kinase inhibitor 1C ; COROA1:coronin actin binding protein 1A; ESR-1: estrogen receptor 1; FOS:FBJ murine osteosarcoma viral oncogene homolog; FOXO3: Forkhead box O3; ICAM3: intercellular adhesion molecule 3; KIT: v-kit Hardy-Zuckerman 4 feline sarcoma viral oncogene homolog, MMP1: matrix metalloproteinase 1; PPP2R2A: protein phosphatase 2 (formerly 2A), regulatory subunit B, alpha isoform, PTEN:phosphatase and tensin homolog;SELE:selectin E;SOD2:superoxide dismutase 2; SSSCA1:Sjogren syndrome/scleroderma autoantigen 1, STAT5A: signal transducer and activator of transcription 5A; TCEAL1:transcription elongation factor A (SII)-like 1; TP53: tumor protein p53

**Figure 5:**
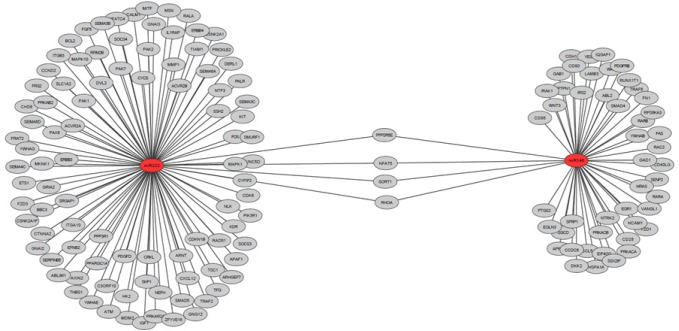
Schematic representation of the general prediction targets for hsa-miR-146 and hsa-miR-222, according to the miRDip available at http://ophid.utoronto.ca/mirDIP

Table 2 shows the main targets of hsa-miR-146b and hsa-miR-222 and their oncogenesis-related functions, according to the results of our search of the literature. The cancer-related pathways that are involved with hsa-miR-146 and hsa-miR-222, according to the miRDip tool, are shown in Figure 4 *C* and D; the targets found using the KEGG algorithm are shown in Figure 5.

## DISCUSSION

To our knowledge, this is the first study to propose a miRNA signature for EBV^+^DLBCLe and to identify hsa-miR-146b and hsa-miR-222 as possible biomarkers and therapeutic targets. The main deregulated routes are the NF-κB, PI3K/AKT pathways and PTEN being a target of the overexpressed hsa-miR-222. Thus, these findings suggest that antagomiRs for hsa-miR-146b and hsa-miR-222 can be used as adjuvant therapy to anthracycline-based chemotherapy (R-CHOP) in EBV^+^DLBCLe, which we confirmed has a poorer prognosis than DLBCL, NOS.

Malumbres and colleagues [22] found that hsa-miR-222 could differentiate the DLBCL into non-GCB or GCB subtypes, and the expression of some of this miRNA was correlated with progression free survival [23] and clinical outcome [24]. Hsa-miR-222 is also associated with immune regulation and other B-cell tumors [25].

We evaluated the expression pattern of miRNAs in EBV^+^DLBCLe and EBV–DLBCL patients aged 50 years or more to establish a signature profile for EBV^+^DLBCLe. This result was consistent with those in the literature, even in studies from other parts of the world. In Asian countries, the prevalence of EBV^+^DLBCLe varies between 8%-12%. The proportion increases with age, reaching 20%-25% in individuals older than 90 years [4].

In available previous studies, the median age was 71 years (range, 45-92 years), and there was a slight male predominance (1.4:1) [4]. Thus, our results were similar to those of previous reports regarding age but different in relation to sex. In our series, only 22% of EBV^+^DLBCLe cases had extranodal involvement. The results in the literature showed variations in extranodal involvement depending on nationality [26]. IPI scores were similar to those expected in the literature [27].

When we compared patients with EBV^+^DLBCLe and EBV–DLBCL, we found no association among clinical variables (age, sex, clinical stage, IPI, or extranodal involvement). Only the presence of B symptoms was significantly higher in EBV–DLBCL patients. A possible explanation for this difference may be due to the interval between the onset of symptoms and seeking for care and diagnosis, since the groups did not differ in stage or IPI, which were both directly related to tumor aggressiveness. Besides, most of the EBV^+^ cases were obtained from the multicenter study and the presence of B symptoms may be not consistently reported.

The morphological analyses revealed that the majority of cases are polymorphic variant (66%) and it is in agreement with previous studies in Western countries [26]. Although elucidation of these morphologic variants is of value to facilitate recognition of EBV^+^DLBCLs, these morphologic variants did not have prognostic relevance [26].

Only 7% of our cases were positive for CD30 and was lower than expected for Western countries [26]. After the recent study of Ok et al. where EBV^+^ and CD30^+^ conferred an inferior outcome for DLBCL treated with R-CHOP, the authors suggest that brentuximab could be used to target CD30 in this patient's subset [26]. LMP1 expression was 62.5% of in our study corresponding to EBV latency pattern type II or III.

According to the existing data, most cases of EBV^+^DLBCLe are non-GCB. Immunophenotypically, cells express CD20. CD10 and Bcl-6 are commonly negative, CD30 is variable, CD15 is negative, and IRF4/MUM1 is often positive [28,29]. There was a statistically significant difference when comparing the EBV^+^ and EBV– groups according to the Hans algorithm (p=0.0472). Therefore, we confirmed the association between tumor origin and EBV expression, as mentioned in other studies [4,30,31] with an important correlation with NF-kappa B activation [20]. Salles algorithm [14] was not able to differentiate the two groups.

We found that EBV^+^DLBCLe patients had poorer survival which is consistent with findings in the literature [4,28,32]. Most of our patients were treated with CHOP without rituximab. This finding is not in agreement with a recent study in Western countries, where all patients were treated with R-CHOP and EBV^+^ did not predict worse prognosis. The authors suggest that rituximab could overcome inferior outcome observed in pre-rituximab era. Survival curves comparing the categories of the other variables showed no statistical difference. EBV^+^ disease may be more indicative of poor prognosis than other known variables, such as high IPI, non-GCB, or Salles algorithm group 3 or 4. This finding reinforces the role of EBV in the pathogenesis and aggressiveness of this disease.

In our cohort, only 13 patients (2 EBV^+^DLBCLe and 11 EBV–DLBCL) were treated with immunotherapy (monoclonal antibody anti-CD20) and R-CHOP, which is currently the first-line treatment for DLBCL. This is because many patients were treated prior to 2010 and DLBCL immunotherapy was established in the public health system in Brazil after 2007.

We evaluated the expression patterns of miRNAs in patients with EBV^+^DLBCLe and EBV–DLBCL using pre-established platforms. The number of selected miRNAs, only seven, is small comparing the two groups, but we considered stringent methods (two endogenous normalizers and two mathematical methods) and we are comparing tumors that are very similar since both groups are DLBCL.

After validation, five miRNAs remained to be valuable to differentiate the two groups: hsa-miR-126, hsa-miR-146a, hsa-miR-146b, hsa-miR-150, and hsa-miR-222. We propose this is the miRNA signature for characterizing EBV^+^DLBCLe cases.

The miRNAs hsa-miR-146b and hsa-miR-222 had high specificity in EBV^+^DLBCLe patients and were considered potential biomarkers for this disease. Two miRNAs were excluded from the final signature: hsa-let-7g because of a lack of statistical significance and hsa-miR-151, which was underexpressed in both groups (not confirming pilot study findings).

In particular, the relative expression of hsa-miR-222 was assessed in more detail because of the observation of two subpopulations in the EBV^+^ group that had overexpression (9 cases) and underexpression (15 cases) of this miRNA. Although we have identified a prevalence of clinical features that are predictive of poor prognosis in cases with increased expression of hsa-miR-222, we found a statistically significant difference only in the Salles classification (p= 0.0245, Fisher's Exact test). This supports the notion that in the EBV^+^DLBCLe subgroup, overexpression of hsa-miR-222 was associated with poor prognosis. Hsa-miR-222 has already been described in another study [9], however underexpressed in EBV^+^DLBCL cases, but it was analyzed by different method (deep sequencing) and the authors did not evaluate specifically the elderly group as in the present study.

We performed a homology blast of the seven human miRNAs selected in this study against 44 virus miRNAs using bioinformatics tools, according to the methods of Babu and colleagues [18]. Surprisingly, the human miRNAs that were homologous to EBV were those with high specificity in the ROC curve. Hsa-miR-146b stem-loop sequence was homologous with EBV-miR-BART3 and hsa-miR-222 stem-loop sequence was homologous with EBV-miR-BART17. The targets of these miRNAs are related to oncogenesis leading us to the hypothesis that the presence of the virus may contribute significantly to pathogenesis of this disease. It is not clear whether we are evaluating the expression of the human miRNA or its viral counterpart. However, the homology is important when the biologically relevant mature 5q sequence is considered (and not only the stem-loop sequence) and it does not happen neither for hsa-miR-146a nor for hsa-miR-222.

There have been few studies of the targets and pathways that are directly involved with hsa-miR-146b. It is known that hsa-miR-146a overexpression is associated with cancer [33,34]. Thus, we reviewed the homologous features between hsa-miR-146a and has-miR-146b using the Needleman-Wunsch algorithm in order to verify similarities for future search for relevant targets and pathways. Although the identity of 44.1% with a high score (147.5) did not completely rule out any association between the two miRNAs, the pattern of expression in our study was different between the graphs of relative expression in EBV^+^DLBCLe and EBV–DLBCL groups.

Finally, used KEGG, miRDip, GeneCards, and GeneRIF to identify the pathways related to cancer and apoptosis as the main targets of the two miRNAs that were possible biomarkers in our study. One miRNA may act in several target mRNAs, affecting multiple signaling pathways [35]. The targets of hsa-miR-146b and its viral counterpart are *INTS6* and IPO, which are a tumor suppressor [21] and a mediator of inflammation, respectively[30,36]. However, the targets of hsa-miR-222 and its viral counterpart, EBV-miR-BART17, were considered extremely relevant: PTEN, CDKN1B (P27), CDKN1C, STAT5A, FOS, KIT, ICAM1, SOD2, and BCLAF1. Thus, once overexpressed, hsa-miR-222 interferes with important proteins related to oncogenesis, cell cycle regulation, cell transcription, cell adhesion, oxidative stress, and apoptosis inhibition [35,36].

Given the importance of hsa-miR-222 in EBV^+^DLBCL, we searched for functional correlation in other types of cancer (lung, breast, and prostate) in which this miRNA was overexpressed. The tumor suppressor, PTEN, blocks the PI3K/AKT pathway and modulates the AKT-mTOR pathway by dephosphorylating compounds such as intrinsic plasma membrane protein 3; when not phosphorylated, these compounds interact through synergism with interacting acting protein 1 to suppress AKT1 activation [37]. It is known that in EBV^+^DLBCLe, this pathway is activated [31]. Therefore, our finding of hsa-miR-222 overexpression is in agreement with the findings of previous reports in this disease, making knockdown of has-miR-222 and stimulation of PTEN possible therapeutic targets.

Another pathway activated in EBV^+^DLBCLe is the NF-κB pathway [31]. ICAM-1, a target of hsa-miR-222, acts in the NF-κ protein in B1A and its inhibitor IκB. Hsa-miR-146a and hsa-miR-126, which are also overexpressed in EBV^+^DLBCLe cases, interact with NF-κB1 and IκBA, respectively [38].

Krützfeldt and colleagues [39] identified a new class of oligonucleotides known as antagomiRs that function as silencers of miRNA expression in mice. These single-stranded RNA molecules of 21-23 nucleotides are conjugated to cholesterol and act in complementarity in the mature miRNA target. They are generally given by intravenous infusion and an effect is noticeable after a week, either by an analysis of miRNA expression or measurement of target proteins that are directly involved with the miRNA. There are experimental studies on obesity in mice that were able to silence the hsa-miR-146b through locked nucleic acid-miR-146b antagomiR without impairing the liver receptor function, and therapy effectiveness was observed by measuring levels of SIRT (sirtuin), a protein with an inverse relationship with miRNA expression [40].

Antisense oligonucleotides conjugated with cholesterol anti-miR-222 were injected into tumors in animal models of prostate cancer. In the literature, it was found that underexpression of this miRNA is associated with increased P27 protein, which is one of its known targets, and saves inverse relationship with the miRNA [41]. Peptide nucleic acid antagomiRs have also been described that act by decreasing the expression levels of hsa-miR-221, which is homologous with has-miR-222, with good responses *in vivo* and subsequent increases in P27 [42]. The results of preclinical studies indicate that these peptide nucleic acid antagomiRs can be used successfully in cancer treatment [43].

Since we consider hsa-miR-146b and hsa-miR-222 to be biomarkers for EBV^+^DLBCLe, the results of this study and the above described models suggest that antagomiRs can be used as adjuvant therapy to the current treatment, R-CHOP.

Further functional studies are needed to evaluate the effect of hsa-miR-222 inhibition in EBV^+^DLBCL cell lines. Inhibition of this miRNA could change the level of certain targets, such as PTEN, that are strongly implicated in regulation of the PI3K/AKT pathway. We could also measure the levels of other proteins, such as P27, or determine whether there is less tumor cell proliferation, as demonstrated in *in vitro* studies of other types of cancer, such as glioblastoma [44].

MiRNAs that were differentially expressed in EBV^+^DLBCLe and EBV–DLBCL patients may affect the pathogenesis of EBV^+^DLBCLe and contribute to poor clinical outcomes via several mechanisms. To our knowledge, we have identified the first miRNA signature profile that characterizes this neoplasm. Our findings show that epigenetic events can be responsible for the poor outcome of EBV^+^DLBCLe patients and suggest future therapeutic modalities.

## METHODS

### Patients

We identified seventy-one patients who were treated at Sao Paulo Hospital between 2000 and 2010 and for whom paraffin blocks were available for immunohistochemical and molecular analyses. All eligible cases corresponded to DLBCL cases. Patients were 50 years or older and thus were classified as EBV^+^DLBCLe (pilot study). We excluded HIV-positive cases and patients with a history of congenital immunodeficiency, lymphoma, post-transplantation lymphoproliferative disease, or primary central nervous system and cutaneous lymphoma. Sixty-nine cases were considered DLBCL not otherwise specified and two were T-cell/histiocyte rich large B-cell lymphoma. Demographic and clinical data, such as age, sex, histological diagnosis, Ann Arbor clinical stage, and International Prognostic Index (IPI), were obtained from our databank and patients' clinical records. We validated the arrays' results in a larger multicenter cohort. The remaining 23 EBV^+^DLBCLe cases, obtained from other centers at Sao Paulo state were joined for this multicenter study. Thus, we compared a total of 94 cases including 29 EBV^+^DLBCLe and 65 EBV^−^DLBCL patients. This study was approved by Ethics Committees of all participating institutions. Due to the retrospective characteristic of the study, the study was classified as minimal or no risk project.

### Tissue microarray (TMA)

Using paraffin blocks (biopsies of lymph nodes or tumor masses), we constructed a tissue microarray (TMA) [10] using Beecher Instruments equipment (Estigen, Tartu, Estonia). Each case in the pilot study (n=71) was evaluated by an experienced pathologist (A.C.A.) for histologic confirmation and to chose the tumoral area to be drilled to construct the TMA block. All H&E-stained DLBCL slides demonstrated more 70% of area tumor, with no significant tissue necrosis. The samples were represented in duplicate in the receptor block.

### *In situ* hybridization

In situ hybridization (ISH) was used to detect EBV (EBV^−^encoded RNA probe, ZytoVision, Bremerhaven, Germany) with the Dako Device (Carpinteria, CA, USA) in a tissue microarray slide. We used 50% positivity as a cut-off for classifying EBV–DLBCLe [11].

### Immunohistochemical analysis

The following markers were assessed to classify cases according to their cellular origin (Hans *et al.* algorithm [12] and Visco/Young algorithm [13] and prognostic importance (Salles *et al.* algorithm [14]): CD10, Bcl-6, MUM-1, Bcl-2, and Ki-67 (three slides of the TMA block for each marker analyzed in duplicate). CD30 and LMP1 were also performed. Slide images were captured using ScanScope AT Turbo equipment (Aperio Technologies, Vista, CA, USA). Slide analyses were performed by two independent observers using a semi-quantitative method (T.A.A. and A.C.A.). The reaction was considered positive according to the cut-offs previously established for each algorithm: 30% or more for CD10, Bcl-6, and MUM-1 markers and 75% or more for Bcl-2 and Ki-67;10% or more for LMP1 and 30% or more for CD30. We considered several technical aspects such as the quality of the material, the correlation between two or more slides, and the median of the three scores, in that order, to classify each sample and minimize intra observation errors.

### MiRNA extraction and global expression

In the pilot and multicenter studies, we obtained tumor tissue samples from 40-80 μm cuts of the paraffin block that contained the diagnostic specimen and placed them in 1.5 mL Eppendorf tubes, in duplicate. Total RNA was obtained from tumor slides using the Recover All total nucleic acid isolation kit (Applied Biosystems, Foster City, CA, USA). Four EBV^+^DLBCLe and four EBV–DLBCL cases were analyzed in this part of the pilot study. We obtained cDNAs using 10ng of total RNA without preamplification and Megaplex Pools for miRNA expression (Applied Biosystems). The cDNA was inserted into two platforms (TaqMan Array Human microRNA A+B Cards) containing 384 human miRNAs each (TaqMan low-density arrays) on 7900 Real Time PCR System (Applied Biosystems). We considered miRNAs to be differentially expressed when the foldchange was above or below 1.5. The normalization method was performed using the endogenous RNU48 identified as the most stable among samples by software Normfinder (http://www.moma.dk/normfinder-software) and RNU6 recommended by the manufacturer, in a comparative way.

### Real-time PCR validation

Real-time quantitative PCR was performed using 7500 Real-Time PCR System (Applied Biosystems) with TaqMan small RNA kit assays (000468, 000473, 0001097, 002228, 002254, 002276, 002282) to validate the pilot study results in a multicenter cohort. Samples were studied in triplicate. The normalization method 2-deltaCt [15,16] was performed using endogenous RNU48 (001006). MiRNAs were considered differentially expressed using the 1.5 cut-off [17].

### Identified pathways and targets of miRNAs

The relevant pathways and targets of miRNAs were identified using the following tools: MiRBase (http://www.mirbase.org), miRDip (http://ophid.utoronto.ca/mirDIP/), GeneCards (http://www.genecards.org), Gene Reference Into Function (GeneRIF) (http://www.ncbi.nlm.nih.gov/gene), Database for Annotation Visualization and Integrated Discovery (DAVID) version 6.7 (http://david.abcc.ncifcrf.gov), and the Kyoto Encyclopedia of Genes and Genomes (KEGG) (http://www.genome.jp/kegg).

### Homologous features between human and EBV miRNA

We performed a homology blast of selected human miRNAs with 44 virus miRNAs, according to the methods of Babu and colleagues [18]. The survey was conducted using the nucleotide sequences of the miRNAs of interest, both human and viral, from the miRBase database (http://www.mirbase.org). With this information, we compared both sequences by scanning them with a homology alignment called the Needleman-Wunsch algorithm, which is available at http://www.ebi.ac.uk/emboss/align/. Two sequences were considered homologous when a score higher than 40 and an identity around 50% are obtained.

### Statistical analysis

The analysis was performed in the mathematical statistical environment “R” (http://www.r-project.org) using non-parametric tests as rank products (RankProd package) and Wilcoxon rank-sum (R-stats). To assess possible clinical differences between the EBV^+^DLBCLe and EBV– groups, we used the chi-square test. We used the Mann-Whitney test to estimate the significance of the medians of relative miRNA expression in these groups. ROC curves were constructed to evaluate the sensitivity, specificity, and positive and negative predictive values of miRNAs that showed different biological behavior between the EBV+DLBCLe and EBV–DLBCL groups. Overall survival (OS) was calculated as the time from the date of diagnosis (date of biopsy) to death. Deaths not related to DLBCL and patients who lost follow-up were censored. Survival curves were constructed according to the Kaplan-Meier method [19] and differences in survival were analyzed using the log-rank and Breslow tests. For all statistical analyses, p <0.05 was considered statistically significant. Statistical analysis, gene expression graphs, and survival curves were created with GraphPad Prism software version 6.0 (http://www.graphpad.com).
